# Transcutaneous electrical acupoint stimulation accelerates gastrointestinal function recovery after abdominal surgery: a systemic review and meta-analysis of randomized controlled trials

**DOI:** 10.1097/JS9.0000000000002946

**Published:** 2025-08-08

**Authors:** Chenwei Wu, Zhenghao Deng, Yabing Zhu, Yi Li, Yuwei Chen, Lin Wang, Jinbao Li, Lina Huang, Qing Tu

**Affiliations:** a School of Anesthesiology, Shandong Second Medical University, Weifang, China; b Department of Anesthesiology, Shanghai General Hospital, Shanghai Jiao Tong University School of Medicine, Shanghai, China

**Keywords:** abdominal surgery, enhanced recovery after surgery, gastrointestinal function, laparoscopic surgery, meta-analysis, transcutaneous electrical acupoint stimulation

## Abstract

**Objective:**

This meta-analysis systematically evaluates the efficacy of transcutaneous electrical acupoint stimulation (TEAS) as an adjunctive therapy for Enhanced Recovery After Surgery (ERAS) protocols in patients undergoing abdominal surgery.

**Methods:**

We systematically searched PubMed, Embase, Ovid, and Web of Science for relevant randomized controlled trials (RCTs) investigating the application of TEAS in laparoscopic surgical procedures, published from database inception to 25 March 2025. Primary outcomes included time to first flatus, defecation, and time to oral intake after surgery. The secondary outcomes including incidence of postoperative nausea and vomiting (PONV), postoperative nausea (PON), postoperative vomiting (POV), pain intensity, length of hospital stay, and total hospitalization costs.

**Results:**

We ultimately included 16 RCTs (comprising 2300 participants) in this meta-analysis. The results demonstrated that TEAS accelerated the time to first exhaust and defecation after abdominal surgery, as well as shortened the time to oral intake of patients received open surgery patients. Additionally, TEAS reduced the incidence of PONV, PON, and POV, alleviated pain intensity, shortened hospital stays, and lowered overall hospitalization costs of patients received abdominal surgery.

**Conclusions:**

The study indicated that TEAS effectively enhanced ERAS outcomes in abdominal surgery patients by accelerating the time to first defecation, flatus, and oral intake, while reducing postoperative pain intensity, PONV incidence, length of hospital stay, and hospitalization costs. These benefits support the clinical adoption of TEAS and improve patient outcomes. However, the observed effects of TEAS should be interpreted in the context of certain limitations.

## Introduction

Recent advances in artificial intelligence (AI) have transformed research methodologies across multiple disciplines. This meta-analysis is compliant with the TITAN Guidelines 2025-governing declaration and use of AI^[1]^.

**HIGHLIGHTS**

- First comprehensive meta-analysis across abdominal surgery types evaluating TEAS on the postoperative gastrointestinal function recovery.
- Providing the first pooled evidence that TEAS shortens length of hospital stay and reduces hospitalization costs, addressing a critical gap in ERAS cost-effectiveness analyses of patients undergoing abdominal surgery.
- Having potential to transform TEAS from an anecdotal adjunct to an evidence-based ERAS component in clinical practice.

Enhanced Recovery After Surgery (ERAS) has emerged as a rapidly evolving surgical paradigm in the past decades. As an evidence-based, multimodal, and multidisciplinary perioperative care pathway, ERAS aims to facilitate fast recovery and reduce surgical stress response in patients undergoing major procedures. This integrated approach encompasses three key phases: preoperative optimization, intraoperative management, and postoperative interventions. Core elements include preoperative patient education, minimized fasting periods, multimodal analgesia, early enteral nutrition, and prompt postoperative mobilization^[2,3]^. Within the framework of multidisciplinary collaboration, anesthesiologists and anesthetic strategies constitute fundamental components of ERAS protocols, and anesthesiologists have formed specialized ERAS teams to strictly implement ERAS programs throughout the entire perioperative period to promote enhanced postoperative recovery^[4]^. Studies have demonstrated that the implementation of ERAS protocols is associated with a marked reduction in postoperative complications, ultimately decreasing in length of hospital stay by approximately 50%^[5-7]^. Multiple evidence-based interventions have been implemented to optimize ERAS protocols, including preoperative carbohydrate loading with electrolyte solutions, multimodal analgesia for postoperative pain management, and early mobilization strategies to accelerate recovery, etc.^[8,9]^.

Abdominal surgery, like gastrointestinal, gynecological, and urological procedures, etc., is a frequently performed intervention in clinical practice for managing both benign and malignant pathologies. However, these procedures are often associate with a series of complications after surgery, particularly gastrointestinal dysfunction^[4]^. ERAS program advocates early oral intake postoperatively, but recovery of gastrointestinal function serves as a prerequisite for oral intake. Early mobilization may facilitate the recovery of postoperative gastrointestinal function^[10]^. In recent decades, despite the widespread clinical adoption of minimally invasive surgery, it is not without certain limitations and challenges. Despite its popularity in abdominal surgery, this approach still significantly affects patients’ postoperative gastrointestinal function^[11]^. Laparoscopic procedure requires the creation of pneumoperitoneum to optimize the surgical field. However, this intervention can induce physiological alterations due to carbo-peritoneum, resulting in acid-base balance, blood gas parameters, and cardiopulmonary function, which may potentially lead to perioperative complications^[12]^. Furthermore, pneumoperitoneum has been shown to suppress gastrointestinal motility, leading to functional impairment manifested by increased intestinal gas accumulation. This pathophysiological alteration predisposes patients to a higher risk of inflammatory responses, infectious complications, and the development of postoperative abdominal distension and intestinal obstruction, ultimately impair the recovery of gastrointestinal function^[13,14]^.

Recent studies advocate non-pharmacological treatments to fast gastrointestinal function recovery postoperatively, which is beneficial for reducing medical costs, minimizing drug-related adverse reactions, and reducing length of hospital stay, etc.^[4]^. Non-pharmacological ERAS measures include preoperative carbohydrate loading, early postoperative oral feeding, multimodal analgesia, physical therapy, psychological support, and personalized nutritional interventions^[15–17]^. Transcutaneous electrical acupoint stimulation (TEAS), as a traditional Chinese acupuncture therapy integrating acupuncture and electrical stimulation, is widely utilized in clinical practice, particularly in East and Southeast Asia^[18]^. Our previous study indicated TEAS is a reasonable modality to incorporate into a multimodal management approach to prevent postoperative nausea and vomiting (PONV) after anesthesia^[19]^. However, no current study has yet assessed the efficacy of TEAS on the recovery of postoperative gastrointestinal function. This meta-analysis aims to evaluate the role of TEAS in enhancing ERAS for patients undergoing abdominal surgery.

## Materials and methods

The meta-analysis work has been reported in line with PRISMA (Preferred Reporting Items for Systematic Reviews and Meta-Analyses) and AMSTAR (Assessing the Methodological Quality of Systematic Reviews) Guidelines^[20,21]^, which was registered in PROSPERO (International Prospective Register of Systematic Reviews, registration number: CRD42024600652).

### Inclusion and exclusion criteria

Two of our authors independently reviewed the eligibilities of the relevant articles based on the following inclusion: (1) article type: randomized controlled trial (RCT). (2) interventions: TEAS applied in experimental group, while sham TEAS in controlled group. (3) RCTs has enrolled the indicators for evaluating gastrointestinal function or ERAS programs as their outcomes. Exclusion criteria including: (1) article types of comments, case reports, crossover studies, letters, editorials, review articles, meta-analysis or retrospective studies; (2) studies involving animal experiments; (3) study data that cannot be extracted or unable to obtain raw data. If there exist any discrepancies, the disagreements can be resolved via discussion meeting or consulting to the corresponding author.

### Search strategy

In the study, the databases of PubMed, Embase, Ovid and Web of Science were searched for relevant randomized controlled trials (RCTs) published from database inception to 25 March 2025 of which focus on elective abdominal surgeries, including but not limited to colorectal resection and gastrectomy, as these procedures are most frequently associated with ERAS protocols and have sufficient data for TEAS intervention analysis. The MeSH was restricted to TEAS (e.g., ‘transcutaneous electrical acupoint stimulation’ OR ‘TEAS’ OR ‘transcutaneous acupoint electrical stimulation’ OR ‘TAES’ OR ‘acustimulation OR’ “acupoint stimulation” OR′electroacupuncture’) and abdominal surgery OR gastrointestinal surgery OR gynecologic surgery OR urological surgery OR laparoscopic surgery, and MeSH relates to ERAS (e.g., ‘enhanced recovery after surgery’ OR ‘ERAS’ OR ‘fast-track surgery’ OR ‘rapid recovery surgery’). There were no restrictions on published date, sex and age of participants, anesthesia status. We searched the above terms in the title, abstracts of potential articles. References of relevant studies were also intensively reviewed.

### Quality evaluation

Two authors independently assessed the quality of included studies based on the Jadad scores^[22]^. The following items were strictly assessed: (1) principle of randomization strategy; (2) description of detailed participants and personnel blinding method; (3) the method of participant withdrawal or dropout protocol. Studies with Jaded score ≥4 points were considered high quality. Additionally, risk of bias of enrolled studies by using the Cochrane Risk of Bias Tool were also evaluated, which including the following items: (1) method of random sequence generation, (2) disclose of allocation concealment, (3) blinding method of participants and personnel, (4) blinding method of outcome assessment, (5) disclose of incomplete outcome, (6) disclose of selective reporting. For studies with missing data, we contacted the corresponding authors for raw data.

### Data extraction

Data extraction was independently performed by two reviewers using a standardized form. Any discrepancies between reviewers were resolved through discussion, and when necessary, a third reviewer was consulted to reach a consensus. The following data were extracted from each included study. The first author’s name, published year, country of the study conducted, sample size in each group, type of surgery, detailed information of TEAS intervention, outcomes of interest. If there is a missing data, we will try to contact the corresponding author for the raw data.

### Statistical analysis

Data analysis was performed using Review Manager (version 5.4). Dichotomous data were evaluated using relative risks (RR) with 95% confidence intervals (CI). The heterogeneity was assessed by chi-square test and *I^2^* statistic with *P*-value of ≤0.10 indicating significant heterogeneity. The Mantel-Haenszel randomized effect model was used. A *P*-value of <0.05 were regarded as statistically significant. Sensitivity analysis was conducted by using method of leave-one-out method to address potential heterogeneity from individual endpoint. Publication bias was assessed by Egger’s test.

## Results

### Literature search

**Figure 1.**
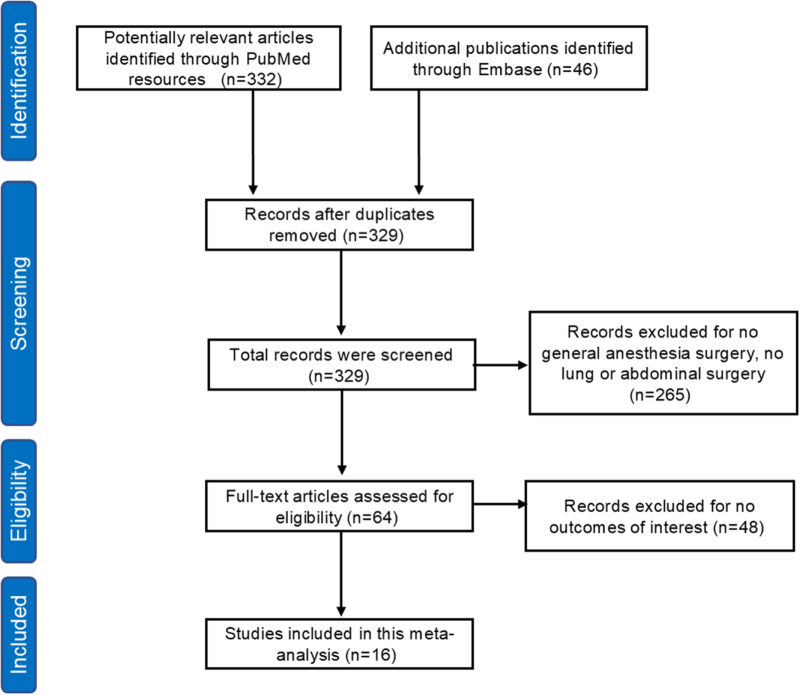
Flow diagram of study selection.

Based on our search strategy, 378 potential studies were initially identified, with 49 duplicates removed. After screening titles or abstracts, 329 studies were excluded. Sixty-four studies were assessed for eligibility, and 48 were excluded after full-text review for irrelevance to the outcomes of interest. Ultimately, 16 studies were finally included in this meta-analysis. The flowchart shows the process of study selection (Fig. 1).

### Characteristics of included studies

The 16 studies^[23–38]^ including a total of 2300 participants, with 1175 participants in the experimental group receiving TEAS and 1125 participants in the control group receiving sham TEAS. Among these included RCTs, most of the studies choose combined acupoints for TEAS. Only one study were conducted in America, the rest were conducted in China. Table 1 summarizes the detailed characteristics of pooled studies. A list of detailed information about TEAS implementation was summarized in Table 2. Table 3 descripted the anatomic location of the target acupoints selected in included studies.

**Table 1:** Characteristics of the included studies

Authors, year	Country	Age	Sample size	Type of surgery	Target outcomes^a^	Jadad scores
Zárate *et al*, 2000	America	≥18	110/56	LC	5,8	6
Pan *et al*, 2023	China	18–60	52/53	Laparoscopic myomectomy myomectomyprocedure	6,9	5
Gao *et al*, 2021	China	≥18	303/307	Colorectal surgery	1,2,3	7
Gu *et al*, 2019	China	?	60/60	LRG	1,2,4,7	7
Huang *et al*, 2019	China	?	29/28	LCR	1,3,8	4
Li *et al*, 2023	China	18–70	79/79	Abdominal surgery	1,2,3,5,6,8	7
Sun *et al*, 2017	China	18–70	95/95	Laparoscopic surgery	1,2	7
Wang *et al*, 2023	China	18–75	40/43	Laparoscopic gastrointestinal tumor surgery	1,4,6,8	7
Wu *et al*, 2023	China	≥18	21/20	PD	1,2,3,	5
Xiong *et al*, 2021	China	<65	31/31	LSG	1,4,6,9	7
Yu *et al*, 2020	China	?	30/30	Laparoscopic gynecological surgery	4,7,8	6
Zhang *et al*, 2018	China	18–65	30/30	LC	1,2,3,7,8	5
Li *et al*, 2021	China	18–70	140/140	Laparoscopic abdominal tumor resection	4,8,9	5
Qin *et al*, 2023	China	18–65	81/81	Laparoscopic gynecological surgery	1,5,8,9	7
Zhou *et al*, 2021	China	18–75	41/40	Colorectal surgery	1,2,4,7,8	5
Li *et al*, 2025	China	25–75	33/32	Colorectal surgery	1,2,3,5,6	6

LC, laparoscopic cholecystectomy LRG, laparoscopic radical gastrectomy; LSG, laparoscopic sleeve gastrectomy; LCR, laparoscopic colorectal resection; PD, pancreaticoduodenectomy

^a^ 1, first-time to exhaust after surgery 2, first-time to defecation after surgery; 3, time to resume diet; 4, incidence of postoperative nausea and vomiting (PONV); 5, incidence of postoperative nausea (PON); 6, incidence of postoperative vomiting (POV); 7, VAS scores; 8, length of hospital stay after surgery; 9, total cost of hospital stay.

**Table 2:** Details of interventions

Authors, year	Time-point of intervention	Acupoints	Frequency
Zárate *et al*, 2000	5-10 min before the end of surgery, lasting for 9 h	P6	3 Hz, 25 mA
Pan *et al*, 2023	30 minutes before the operation and lasting until the end of anesthesia.	PC6, ST36, SP6, ST36	Dense-disperse frequency, 2/100 Hz, intensity of tolerable level
Gao *et al*, 2021	Three times lasting for 3d after surgery, for 30 min	ST36, ST37, SP6	2-10 Hz,8 −15 mA, intensity of feeling de qi sensation.
Gu *et al*, 2019	30 min before induction until 30 min after surgery, three times daily. within 2d after surgery	ST36, PC6	Dense-disperse frequency,2/100 Hz, intensity of feeling de qi sensation
Huang *et al*, 2019	30 min before the induction until the end of surgery	ST 36	Dense-disperse frequency,2/10 Hz, intensity of tolerable level
Li *et al*, 2023	Once a day, from the first day to gastrointestinal function recovery, lasting for 30 min	ST21, SP 15	2-5 Hz, intensity of tolerable level
Liu *et al*, 2021	30 min before the induction to the end of surgery	PC6, LI4, ST36	2/100 Hz, intensity of tolerable level
Sun *et al*, 2017	30 min each time in perioperative period	LI4, P6	Dense-disperse frequency,2/100 Hz, intensity of tolerable level
Wu *et al*, 2023	Twice daily for 1 h and continued for the initial 7 postoperative days	ST-36, PC-6	25 Hz,5 Ma, continuous stimulation for 2 s, stop for 3 s, pulse width 0.5 ms
Xiong *et al*, 2021	30 min before induction to the end of surgery, within 12 h after the surgery, twice daily	PC6, ST36	Dense-disperse frequency,2/10 Hz, intensity of tolerable level
Yu *et al*, 2020	30 min before anesthesia	GV20, EX-HN3, ST36, PC6	Dense-disperse frequency,2/100 Hz,12-15 mA, intensity of tolerable level
Zhang *et al*, 2018	30 min, twice daily, lasting for 4 days, from surgical day to 3 days after surgery	ST36, PC6	ST36:25 Hz,2-10 mA,PC6: 100 Hz,2-10 mA, intensity of tolerable level
Li *et al*, 2021	30 min before surgery till the end of the surgery, and once daily in 3 days after surgery	LI4, PC6, ST36, ST37	2/100 Hz, 2 Hz for 0.6 ms and 100 Hz for 0.2 ms, intensity of tolerable level
Qin *et al*, 2023	30 min, before surgery	PC6, LI4	2/100 Hz,1-12 mA, intensity of tolerable level
Zhou *et al*, 2021	30 min, twice daily in 3 days after surgery	LI4, PC6, BL21, BL27, ST36	20/100 Hz, intensity of tolerable level
Li *et al*, 2025	From the day before surgery, 30 minutes before the start of anesthesia induction, at the start of skin incision, and at the end of surgery to the 1-3 postoperative days, lasting for 30 min	LI4, PC6, ST36, ST37, SP6, ST37	Sparse and dense waves (2/10 Hz), and the intensity of current was adjusted accordingly

**Table 3:** Description list of acupoints

Neiguan (P6)	On the palmar side of the forearm and on the line connecting Quze (PC3) and Daling (PC7), 2 cun above the crease of the wrist.
Zusanli (ST36)	Three cun below Dubi (S 35), one finger breadth from the anterior crest of the tibia.
Hegu (L14)	On the dorsum of the hand, between the first and second metacarpal bones, approximately in the middle of the second metacarpal bone on the radial side, line up the position the transverse crease of the first joint of the thumb with the margin of the web between the thumb and the index finger of the other hand.
Tianshu (ST25)	2 inches apart from the anterior midline in the abdomen, transverse to the navel
Shangjuxu (ST37)	On the anterior aspect of the leg, on the line connecting ST35 with ST41, 6 cun inferior to ST35.
Sanyinjiao (SP6)	On the medial side of the leg, 3 cun above the tip of the medial malleolus, posterior to the medial border of the tibia.
Neiguan (PC6)	On the anterior aspect of the forearm, between the tendons of the palmaris longus and the flexor carpi radialis, 2 cun proximal to the palmar wrist crease.
Liangmen (ST 21)	4 inches above the navel, 2 inches from the anterior midline
Daheng (SP 15)	4 inches next to the navel
Baihui (GV20)	On the midline of the head, about five fingers away from the midline of the front hairline
Yintang (EXHN3)	In the middle of the two eyebrows
Weishu (BL21)	In the upper back region, at the same level as the inferior border of the spinous process of the 12th thoracic vertebra (T12), 1.5 cun lateral to the posterior median line.
Xiaochangshu (BL27)	In the sacral region, at the same level as the first posterior sacral foramen, and 1.5 cun lateral to the median sacral crest.

### Risk of bias assessment and sensitivity analysis

**Figure 2.**
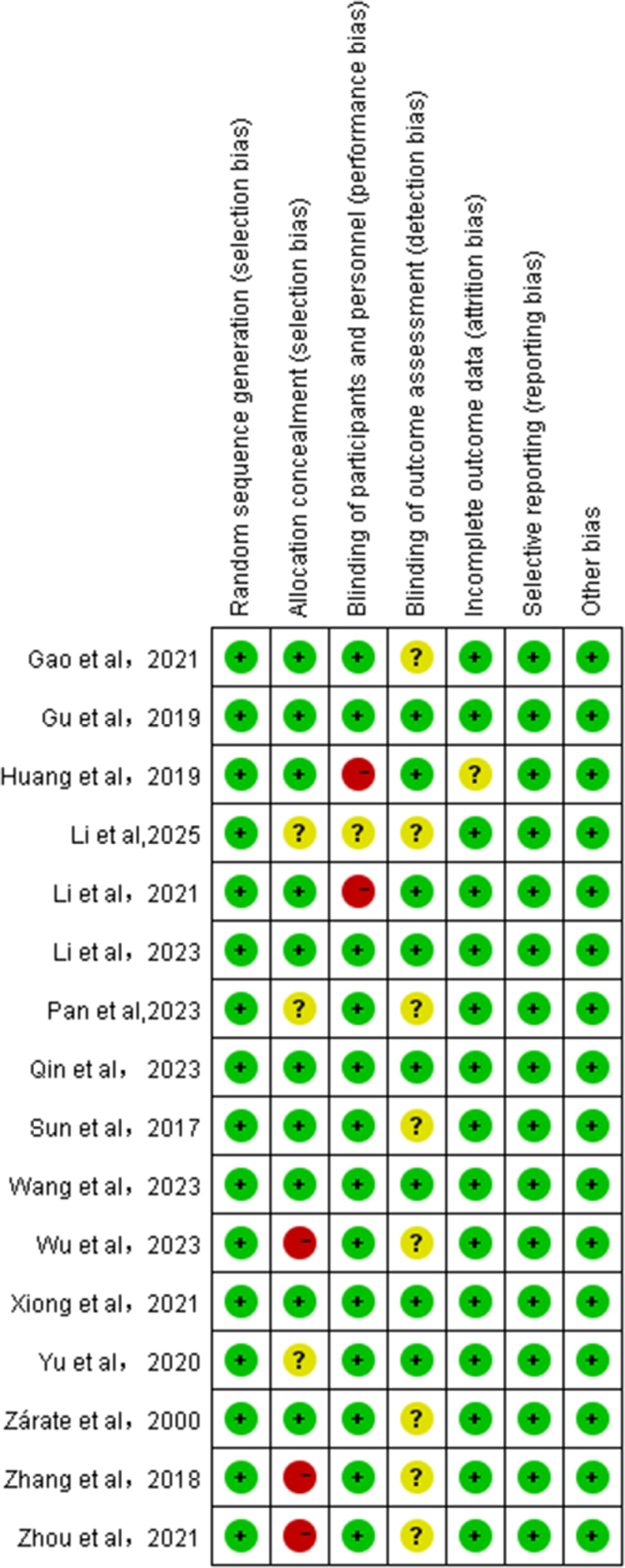
Potential risk of bias of each included study.

Note: “+” represents low risk; “?”represents unclear risk; “-” represents high risk.

The risk of bias of the 16 enrolled RCTs is shown in Figure 2. All the included RCTs described detailed information on the generation of random sequences through random number tables, balanced block randomization, or software generated random numbers. In terms of allocation concealment, 10 RCTs disclosed detailed descriptions of allocation concealment methods, while 6 RCT did not or unclearly provide information of allocation concealment. Thirteen RCTs strictly followed the double-blind method, while three studies did not include or unclearly mention the method of blinding. Regarding the blinding method for outcome assessment, 8 studies included detailed methods for outcomes assessment, while the methods of the other 8 were unclear. Both bias of selective reporting and other bias were considered low risks of included studies. Furthermore, the majority of the included studies demonstrated Jadad scores ≥4, reflecting a high methodological quality (Table 1).

### Publication bias assessment

**Figure 3.**
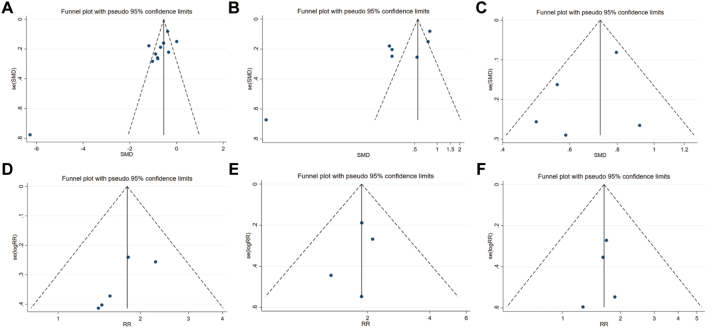
Funnel plots of the outcomes of interests. (A) funnel plot of the first time to first to exhaust after surgery; (B) funnel plot of the first time to first to defecation after surgery; (C) funnel plot of the time to oral intake resumption after surgery; (D) funnel plot of the incidence of PONV; (E) funnel plot of the incidence of PON; (F) funnel plot of the incidence of POV. PONV, postoperative nausea and vomiting; PON, postoperative nausea; POV, postoperative vomiting.

Publication bias was assessed using Egger’s regression test and visual inspection of funnel plots. Egger’s regression test based on the first time to exhaust after surgery (Fig. 3A). The *P* value was 0.014, indicating a publication bias existed. So, we applied the trim-and-fill method to estimate the potential effect of missing studies. The adjusted effect size remained statistically significant (*P* < 0.05), further supporting the validity of our findings. The Egger’s regression test based on the first time to defecation after surgery, in line with the above results, the adjusted effect size was also statistically significant (*P* < 0.05), indicating that the impact of publication bias on the meta-analysis results was minimal (Fig. 3B). While the Egger’s regression test based on the first time to oral intake (*P* = 0.317, Figure 3C), incidence of PONV (*P* = 0.095, Figure 3D), PON (*P* = 0.550, Figure 3E), as well as POV (*P* = 0.608, Figure 3F), indicated no publication bias existed.

### Meta-regression

**Figure 4.**
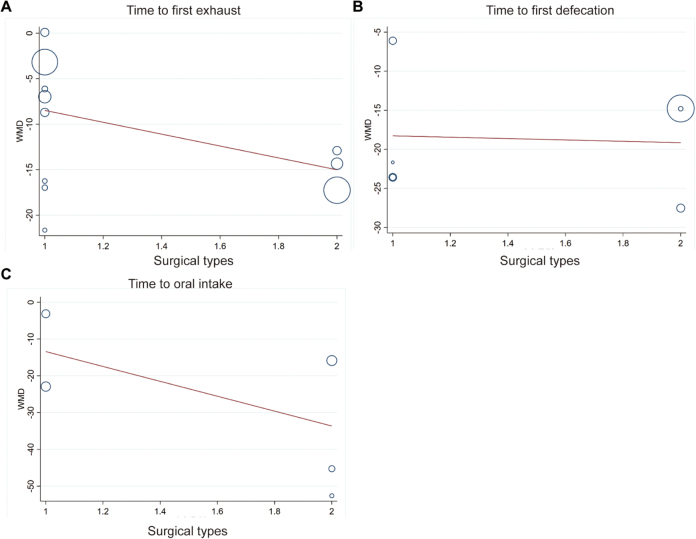
Meta-regression was conducted based on primary outcomes, including (A) time to first exhaust, (B) defecation and (C) oral intake after surgery.

Meta-regression serves as a key methodological approach for investigating potential sources of heterogeneity in meta-analyses. Since we included different kind of surgical type (but all were abdominal surgeries), the meta-regression was conducted by using Stata software based on primary outcomes, including time to first exhaust, defecation and oral intake after surgery. The results found that the type of surgery did not significantly influence the overall results (time to first exhaust, *P* = 0.729; time to first defecation, *P* = 0.148; time to oral intake, *P* = 0.815). Figure 4.

### TEAS accelerates the time to first exhaust after surgery

**Figure 5.**
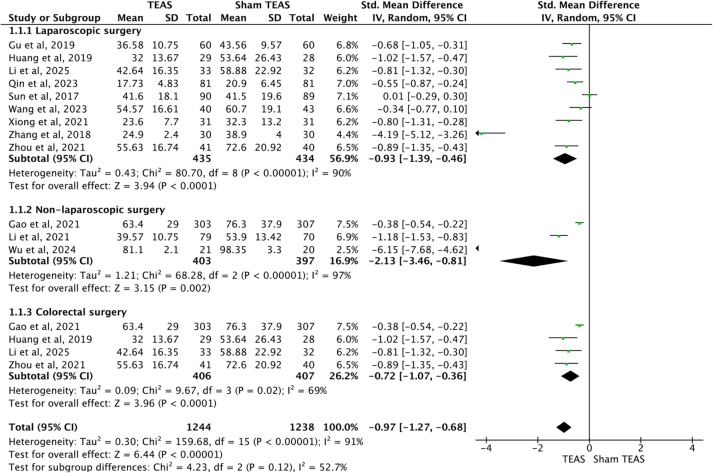
Forest plots comparing the time to first to exhaust after surgery between TEAS and Sham TEAS groups. TEAS, transcutaneous electrical acupoint stimulation.

Eleven studies^[25–28,30–34,37,38]^ recorded the time to first exhaust after surgery, including 9^[26,27,30–32,34,36–38]^ involving laparoscopic surgery and 3^[25,28,33]^ involving non-laparoscopic surgery. For heterogeneity existed among the studies, the random-effects model was performed to analyze the outcome. The results indicated that TEAS accelerated the time to first exhaust after surgery, both in laparoscopic surgery [SMD = −0.93, 95%CI (−1.39, −0.46), *P* < 0.0001] and non-laparoscopic surgery [SMD = −2.13; 95% CI (−3.46, −0.81), *P* = 0.002]. Subgroup analysis based on colorectal surgery^[25,27,37,38]^ indicated that TEAS accelerated the time to first exhaust after surgery [SMD = −0.72, 95%CI (−1.07, −0.36), *P* < 0.0001] (Fig. 5).

### TEAS accelerates the time to first defecation after surgery

**Figure 6.**
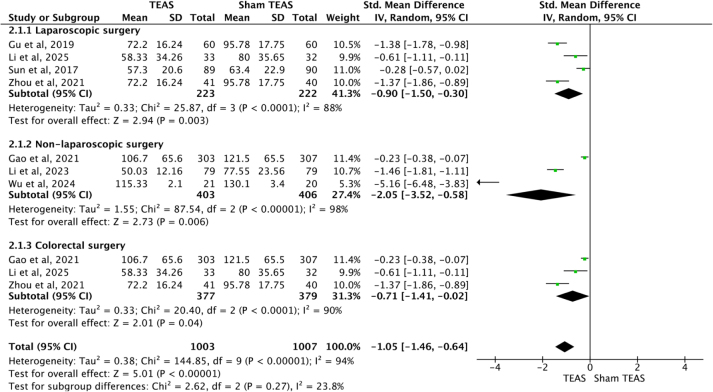
Forest plots comparing the time to first defecation after surgery between TEAS and Sham TEAS groups. TEAS, transcutaneous electrical acupoint stimulation.

Seven studies^[25,26,29,31,33,37,38]^ recorded the time to first defecation after surgery, including 4^[26,31,37,38]^ involving laparoscopic surgery, as well as 3^[25,29,33]^ involving non-laparoscopic surgery. There was also heterogeneity existed among the studies. The random-effects model was performed to analyze the outcome. The results indicated that TEAS accelerated the time to first defecation after laparoscopic surgery [SMD = −0.90, 95%CI (−1.50, −0.30), *P* = 0.003], as well as non-laparoscopic surgery [SMD = −2.05 95%CI (−3.52, −0.58), *P* = 0.006]. Subgroup analysis based on colorectal surgery^[25,37,38]^ indicated that TEAS accelerated the time to first defecation after surgery [SMD = −0.71, 95%CI (−1.41, −0.02), *P* = 0.04] (Fig. 6).

### TEAS accelerates the time to oral intake resumption

**Figure 7.**
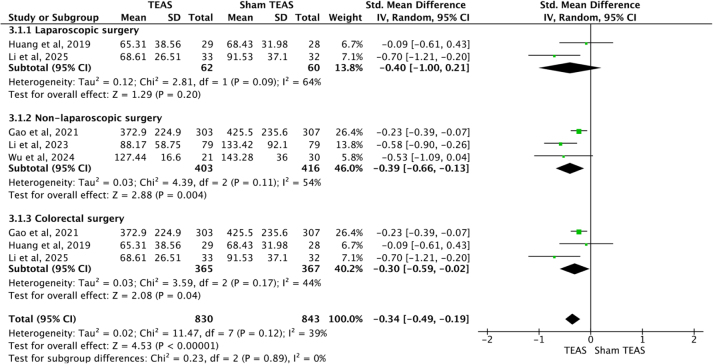
Forest plots comparing the time to oral intake resumption after surgery between TEAS and Sham TEAS groups. TEAS, transcutaneous electrical acupoint stimulation.

Five studies^[25,27,29,33,38]^ documented the time of patients resume their diet, 2^[27,38]^ involving laparoscopic surgery and 3^[25,29,33]^ involving non-laparoscopic surgery. For heterogeneity existed, the random-effects model was used to analyze the results. It seemed that TEAS accelerated the time to resume diet for patients received non-laparoscopic surgery [SMD = −0.39, 95%CI (−0.66, −0.13), *P* = 0.004], but not for laparoscopic surgery [SMD = −0.40, 95%CI (−1.00, 0.21), *P* = 0.20]. Subgroup analysis based on colorectal surgery^[25,27,38]^ indicated that TEAS accelerated the time to oral intake after surgery [SMD = −0.30, 95%CI (−0.59, −0.02), *P* = 0.04]. (Fig. 7).

### TEAS reduced the incidence of PONV, PON and POV

**Figure 8.**
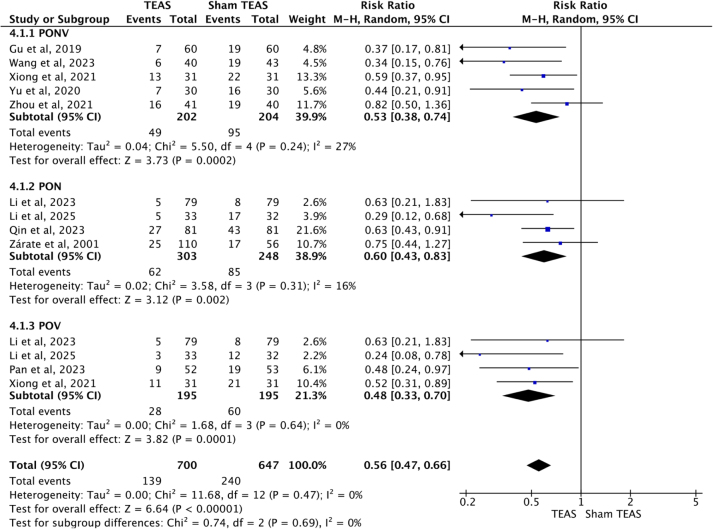
Forest plots comparing the incidence of PONV, PON and POV after surgery between TEAS and Sham TEAS groups. TEAS, transcutaneous electrical acupoint stimulation.

Due to the limited studies included, subgroup analysis was not conducted. For this part, 5 studies recorded the incidence of PONV^[26,32,34,35,37]^ after surgery. No heterogeneity existed. The result indicated the application of TEAS effectively reduced the incidence of PONV [RR = 0.53, 95%CI (0.38, 0.74), *P* = 0.0002]. While 4 studies^[23,29,30,38]^ recorded the incidence of PON, as well as 4^[24,29,34,38]^ recorded the incidence of POV. The results shown that TEAS also reduced the incidence of POV [RR = 0.60, 95%CI (0.43, 0.83), *P* = 0.002], as well as incidence of PON [RR = 0.48, 95%CI (0.33, 0.70), *P* = 0.0001]. (Fig. 8).

### TEAS reduced the VAS scores after surgery

**Figure 9.**
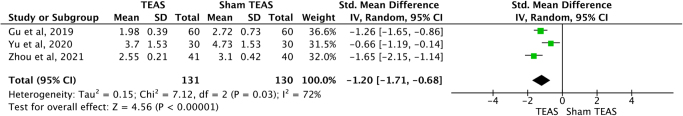
Forest plots comparing the VAS scores after surgery between TEAS and Sham TEAS groups. TEAS, transcutaneous electrical acupoint stimulation.

The pain intensity is also a main factor affecting the recovery of surgical patients. Three included studies^[26,35,37]^ recorded the VAS score after surgery. The result demonstrated that TEAS effectively reduced VAS scores after surgery [SMD = −1.20, 95%CI (−1.71, −0.68), *P* < 0.00001] (Fig. 9).

### TEAS reduced the length and total cost of hospital stay

**Figure 10.**
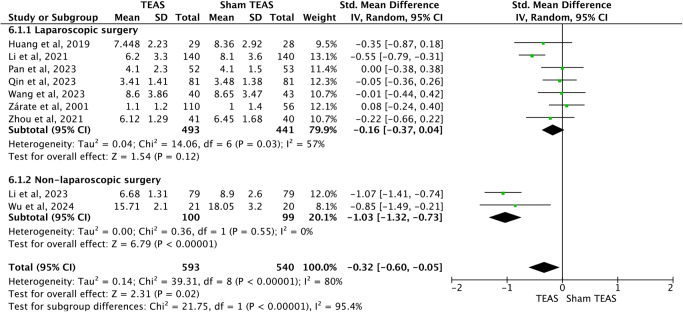
Forest plots comparing the length of hospital stay after surgery between TEAS and Sham TEAS groups. TEAS, transcutaneous electrical acupoint stimulation.

Nine studies^[23,24,27–30,32,33,37]^ recorded length of hospital stay. Two^[29,33]^ were non-laparoscopic surgeries, 7^[23,24,27,28,30,32,37]^ were laparoscopic surgeries. Since heterogeneity existed. We used random-effects model to analyze the outcome. The results of subgroup analysis indicated that TEAS reduced the length of hospital stay after non-laparoscopic surgery [SMD = −1.03, 95%CI (−1.32, −0.73), *P <* 0.00001], but not for laparoscopic surgery [SMD = −0.16, 95%CI (−0.37, 0.04), *P* = 0.12]. But the overall result indicated TEAS may help to shorten the length of hospital stay [SMD = −0.32, 95%CI (−0.60, −0.05), *P* = 0.02] (Fig. 10).

**Figure 11.**
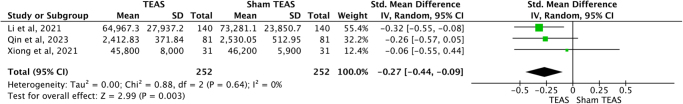
Forest plots comparing the total cost of hospital stay after surgery between TEAS and Sham TEAS groups. TEAS, transcutaneous electrical acupoint stimulation.

Only 3 studies^[28,30,34]^ recorded the total cost of hospital. The total cost of patients in TEAS group was lower than that in control group [SMD = −0.27, 95%CI (−0.44, −0.09), *P* = 0.003] (Fig. 11).

## Discussion

The present meta-analysis provides evidence that TEAS improves postoperative gastrointestinal recovery and reduces adverse events in patients undergoing abdominal surgery. Furthermore, TEAS implementation was also associated with decreased the length and overall costs of hospitalization. These collective findings strongly support the integration of TEAS as an effective adjunctive therapy to ERAS protocols in abdominal perioperative care.

The systematic implementation of ERAS protocols has been demonstrated to significantly enhance surgical safety while simultaneously reducing both length of hospitalization and overall costs. Multiple studies have established TEAS is an effective measure for analgesia, sedation and inhibiting surgical complication, including PONV, emergence agitation^[39–41]^. A recent meta-analysis has assessed the effectiveness for gastrointestinal function improvement^[42]^. However, this analysis only included laparoscopic surgery and only 7 studies were included. Actually, non-laparoscopic surgery maybe more popular for laparoscopic surgery recently. In the study, we included both laparoscopic and non-laparoscopic surgery. Finally, total of 16 studies were included in this meta-analysis, most of studies were conducted in China, only 1 was conducted in America. The Jadad scores of most included studies>5 points, indicating a good quality of included studies. The ERAS protocol emphasizes rapid recovery of gastrointestinal function for patients undergoing abdominal surgery^[42]^. First times to postoperative exhaust and defecation after surgery serves as key clinical indicators of gastrointestinal function recovery^[43,44]^. The results indicated that TEAS shorten the time to first exhaust and defecation. This potential mechanism may owe to TEAS modulates the autonomic nervous system by balancing sympathetic and parasympathetic activity, thereby promoting gastrointestinal motility and reducing postoperative ileus^[45]^. Another study suggested that electroacupuncture reduces visceral sensitivity by regulating the endogenous cannabinoid system^[46]^. TEAS has also been shown to reduce systemic inflammatory responses by downregulating pro-inflammatory cytokines (e.g., IL-6, TNF-α) and upregulating anti-inflammatory mediators, thereby mitigating inflammation-induced gastrointestinal dysfunction^[47]^. The restoration of gastrointestinal function serves as a critical prerequisite for postoperative dietary recovery. Compared to sham TEAS, the time to oral intake resumption was earlier in those received TEAS. Therefore, the above evidence may elucidate the mechanisms of TEAS in improving gastrointestinal function. Our previous study also shown that TEAS helped to reduce the incidence, as well as intensity of PONV, even though TEAS on single acupoint, like PC6^[19]^. The results of the current meta-analysis also indicated TEAS reduced the incidence of PONV, PON, as well as POV after abdominal surgery.

Length of hospitalization and associated costs represent critical outcome measures in ERAS protocols. Our meta-analysis revealed that TEAS significantly reduced both hospitalization duration and total costs compared to sham TEAS controls. This cost-benefit advantage likely stems from TEAS-induced acceleration of gastrointestinal functional recovery, improved pain management, and decreased complication rates—all key determinants of discharge readiness of those received abdominal surgery patients.

Despite demonstrating significant clinical benefits, several methodological limitations warrant consideration in interpreting the findings. Firstly, we only enrolled studies of abdominal surgery. Other types of surgery may also affect postoperative gastrointestinal function recovery, such as central intestinal paralysis after neurosurgery^[48]^. Secondly, most of the study were conducted in China, there may be publication bias existed, the results may not be applicable to all populations. Thirdly, the detailed implementation is various from study to study, lacking unified standard for the specific operation of TEAS. Additional studies are needed to determine an optimal implementation for abdominal surgery. Fourthly, for the included studies, they chose different target acupoints, there is no unified standard for acupoints selection for abdominal surgery, and it is more likely to be based on clinical experience. So, the selection of acupoints should be unified, at least for the same type of surgery. Additionally, given the demonstrated therapeutic benefits, TEAS has the potential to emerge as a perioperative intervention within ERAS protocols, the future studies should highlight the need for standardized reporting of ERAS components in future studies to facilitate more comprehensive analysis of potential confounding factors. When we included studies in our analysis, our search specifically targeted the application of TEAS in abdominal surgeries, not all of which strictly adhered to ERAS protocols. So, given that the extensive and heterogeneous components of ERAS strategies, conducting subgroup analyses based on ERAS implementation would not be methodologically appropriate in this meta-analysis. Considering the varying types of surgical trauma, we only performed subgroup analyses based on surgical approaches (laparoscopic versus non-laparoscopic surgeries). We agree that future studies should include detailed documentation of ERAS protocol elements, and regarding the need for a multicenter study to validate our findings more broadly.

## Conclusion

The results of the meta-analysis demonstrated that TEAS significantly enhances postoperative gastrointestinal function, reduced the incidence of postoperative complications, shortened the length and total cost of hospital stay of patients undergoing abdominal surgery, which may help to reduce waste of medical resources, lower the medical costs, and improve the medical quality in clinical practice. However, the observed effects of TEAS should be interpreted in the context of limitations existed.

## Data Availability

This is a meta-analysis article; all the data is availability in the results of the paper.

## Research registration unique identifying number (UIN)

The meta-analysis was registered in PROSPERO (International Prospective Register of Systematic Reviews, registration number: CRD42024600652; https://www.crd.york.ac.uk).

## References

[1] Agha RA Mathew G Rashid R. Transparency in the reporting of Artificial IN telligence-the TITAN guideline. Prem J Sci 2025;10:100082.

[2] Ljungqvist O Scott M Fearon KC. Enhanced recovery after surgery: a review. JAMA Surg 2017;152:292–98. 28097305 10.1001/jamasurg.2016.4952

[3] Raeder JC White PF. Enhanced recovery after surgery (ERAS): guidelines are important but proper implementation is essential. J Clin Anesth 2022;80:110882. 35597004 10.1016/j.jclinane.2022.110882

[4] Sun Y Liang X Chai F Shi D Wang Y. Goal-directed fluid therapy using stroke volume variation on length of stay and postoperative gastrointestinal function after major abdominal surgery-a randomized controlled trial. BMC Anesthesiol 2023;23:397. 38049713 10.1186/s12871-023-02360-1 PMC10694978

[5] Spanjersberg WR Reurings J Keus F. Fast track surgery versus conventional recovery strategies for colorectal surgery. Cochrane Database Syst Rev 2011;2:CD007635. 21328298 10.1002/14651858.CD007635.pub2 PMC13061361

[6] Stone AB Grant MC Pio Roda C. Implementation costs of an enhanced recovery after surgery program in the united states:a financial model and sensitivity analysis based on experiences at a quaternary academic medical center. J Am Coll Surg 2016;222:219–25. 26774492 10.1016/j.jamcollsurg.2015.11.021

[7] Thiele RH Rea KM Turrentine FE. Standardization of care: impact of an enhanced recovery protocol on length of stay, complications, and direct costs after colorectal surgery. J Am Coll Surg 2015;220:430–43. 25797725 10.1016/j.jamcollsurg.2014.12.042

[8] Ljungqvist O de Boer HD Balfour A. Opportunities and challenges for the next phase of enhanced recovery after surgery: a review. JAMA Surg 2021;156:775–84. 33881466 10.1001/jamasurg.2021.0586

[9] Engelman DT Ben Ali W Williams JB. Guidelines for perioperative care in cardiac surgery: enhanced recovery after surgery society recommendations. JAMA Surg 2019;154:755–66. 31054241 10.1001/jamasurg.2019.1153

[10] Castelino T Jf F Jr Niculiseanu P. The effect of early mobilization protocols on postoperative outcomes following abdominal and thoracic surgery: a systematic review. Surgery 2016;159:991–1003. 26804821 10.1016/j.surg.2015.11.029

[11] Oti C Mahendran M Sabir N. Anaesthesia for laparoscopic surgery. Br J Hosp Med (Lond) 2016;77:24–28. 26903452 10.12968/hmed.2016.77.1.24

[12] Safran DB Orlando R3. Physiologic effects of pneumoperitoneum. Am J Surg 1994;167:281–86. 8135322 10.1016/0002-9610(94)90094-9

[13] McCready JE Gozzard H Tisotti T. Effect of pneumoperitoneum on gastrointestinal motility, pain behaviors, and stress biomarkers in guinea pigs (Cavia porcellus). Am J Vet Res 2022;83:ajvr. 10.2460/ajvr.22.01.0001 35895798

[14] Makanyengo SO Carroll GM Goggins BJ. systematic review on the influence of tissue oxygenation on gut microbiota and anastomotic healing. J Surg Res 2020;249:186–96. 31986361 10.1016/j.jss.2019.12.022

[15] Svensson-Raskh A Schandl A Holdar U. “I have everything to win and nothing to lose”: patient experiences of mobilization out of bed immediately after abdominal surgery. Phys Ther 2020;100:2079–89. 32941610 10.1093/ptj/pzaa168 PMC7720638

[16] Wang Y Xu J Bao R. Massage for gastrointestinal function among participants after abdominal surgery: a protocol for systematic review and meta-analysis. Medicine (Baltimore) 2021;100:e28087. 34889259 10.1097/MD.0000000000028087 PMC8663846

[17] De Felice F Malerba S Nardone V. Progress and challenges in integrating nutritional care into oncology practice: results from a national survey on behalf of the nutrionc research group. Nutrients 2025;17:188. 39796623 10.3390/nu17010188 PMC11722632

[18] Szmit M Krajewski R Rudnicki J. Application and efficacy of transcutaneous electrical acupoint stimulation (TEAS) in clinical practice: a systematic review. Adv Clin Exp Med 2023;32:1063–74. 37026972 10.17219/acem/159703

[19] Chen J Tu Q Miao S. Transcutaneous electrical acupoint stimulation for preventing postoperative nausea and vomiting after general anesthesia: a meta-analysis of randomized controlled trials. Int J Surg 2020;73:57–64. 31704425 10.1016/j.ijsu.2019.10.036

[20] Shea BJ Reeves BC Wells G. AMSTAR 2: a critical appraisal tool for systematic reviews that include randomised or non-randomised studies of healthcare interventions, or both. Bmj 2017;358:j4008. 28935701 10.1136/bmj.j4008 PMC5833365

[21] Page MJ McKenzie JE Bossuyt PM. The PRISMA 2020 statement: an updated guideline for reporting systematic reviews. Int J Surg 2021;88:105906. 33789826 10.1016/j.ijsu.2021.105906

[22] Clark HD Wells GA Huët C. Assessing the quality of randomized trials: reliability of the Jadad scale. Control Clin Trials 1999;20:448–52. 10503804 10.1016/s0197-2456(99)00026-4

[23] Zárate E Mingus M White PF. The use of transcutaneous acupoint electrical stimulation for preventing nausea and vomiting after laparoscopic surgery. Anesth Analg 2001;92:629–35. 11226090 10.1097/00000539-200103000-00014

[24] Pan Y Shao Y Chi Z Jin S Wang J. Transcutaneous Electrical Acupoint Stimulation Accelerates the Recovery of Patients Undergoing Laparoscopic Myomectomy: a Randomized Controlled Trial. J Pain Res 2023;16:809–19. 36925621 10.2147/JPR.S399249 PMC10013582

[25] Gao W Li W Yan Y. Transcutaneous electrical acupoint stimulation applied in lower limbs decreases the incidence of paralytic ileus after colorectal surgery: a multicenter randomized controlled trial. Surgery 2021;170:1618–26. 34497027 10.1016/j.surg.2021.08.007

[26] Gu S Lang H Gan J. Effect of transcutaneous electrical acupoint stimulation on gastrointestinal function recovery after laparoscopic radical gastrectomy-A randomized controlled trial. Eur J Integr Med 2019;26:11–17.

[27] Huang W Long W Xiao J. Effect of electrically stimulating acupoint, Zusanli (ST 36), on patient’s recovery after laparoscopic colorectal cancer resection: a randomized controlled trial. J Tradit Chin Med 2019;39:433–39. 32186016

[28] Li WJ Gao C An LX. Perioperative transcutaneous electrical acupoint stimulation for improving postoperative gastrointestinal function: a randomized controlled trial. J Integr Med 2021;19:211–18. 33495134 10.1016/j.joim.2021.01.005

[29] Li H Wen Q Hu HQ. Transcutaneous electrical acupoint stimulation combined with electroacupuncture for rapid recovery after abdominal surgery: a randomized controlled trial. Zhongguo Zhen Jiu 2023;43:135–40. 36808505 10.13703/j.0255-2930.20220505-0002

[30] Qin J Ye X Ye C. The effect of transcutaneous electrical acupoint stimulation on high-risk patients with PONV undergoing laparoscopic gynecologic surgery: a randomized controlled trial. J Clin Med 2023;12:1192. 36769839 10.3390/jcm12031192 PMC9917901

[31] Sun K Xing T Zhang F. Perioperative transcutaneous electrical acupoint stimulation for postoperative pain relief following laparoscopic surgery: a randomized controlled trial. Clin J Pain 2017;33:340–47. 27437568 10.1097/AJP.0000000000000400

[32] Wang J Lu FF Ge MM. Transcutaneous electrical acupoint stimulation improves postoperative sleep quality in patients undergoing laparoscopic gastrointestinal tumor surgery: a prospective, randomized controlled trial. Pain Ther 2023;12:707–22. 36928500 10.1007/s40122-023-00493-2 PMC10199983

[33] Wu XD Yan HJ Xu YM. Effect and mechanism of needleless transcutaneous neuromodulation on gastrointestinal function after pancreaticoduodenectomy. Scand J Gastroenterol 2024;59:133–41. 37752679 10.1080/00365521.2023.2261060

[34] Xiong Q Min S Wei K. Correction to: transcutaneous electrical acupoint stimulation combined with dexamethasone and tropisetron prevents postoperative nausea and vomiting in female patients undergoing laparoscopic sleeve gastrectomy: a prospective, randomized controlled trial. Obes Surg 2021;31:4669. 34342802 10.1007/s11695-021-05635-z

[35] Yu X Zhang F Chen B. The effect of TEAS on the quality of early recovery in patients undergoing gynecological laparoscopic surgery: a prospective, randomized, placebo-controlled trial. Trials 2020;21:43. 31915045 10.1186/s13063-019-3892-4 PMC6951027

[36] Zhang B Zhu K Hu P. Needleless transcutaneous neuromodulation accelerates postoperative recovery mediated via autonomic and immuno-cytokine mechanisms in patients with cholecystolithiasis. Neuromodulation 2019;22:546–54. 30277014 10.1111/ner.12856

[37] Zhou X Cao SG Tan XJ. Effects of transcutaneous electrical acupoint stimulation (teas) on postoperative recovery in patients with gastric cancer: a randomized controlled trial. Cancer Manag Res 2021;13:1449–58. 33603487 10.2147/CMAR.S292325 PMC7886100

[38] Li X Kou Z Liu R. Transcutaneous electrical acupoint stimulation improves postoperative nutrition and promotes early recovery of gastrointestinal function in patients with colorectal cancer. Comb Chem High Throughput Screen 2025;28:64–73. 39957303 10.2174/0113862073255619231102112544

[39] Jiang M Wang B Liu M. Effect of transcutaneous electrical acupoint stimulation on extubation-related stress response in noncardiac surgery patients: a systematic review and meta-analysis of randomized controlled trials. J Perianesth Nurs 2024;39:990–98. 38904602 10.1016/j.jopan.2024.01.015

[40] Yan W Kan Z Yin J. efficacy and safety of transcutaneous electrical acupoint stimulation (teas) as an analgesic intervention for labor pain: a network meta-analysis of randomized controlled trials. pain ther 2023;12:631–44. 36934401 10.1007/s40122-023-00496-z PMC10199978

[41] Chen J Zhang Y Li X. Efficacy of transcutaneous electrical acupoint stimulation combined with general anesthesia for sedation and postoperative analgesia in minimally invasive lung cancer surgery: a randomized, double-blind, placebo-controlled trial. Thorac Cancer 2020;11:928–34. 32062864 10.1111/1759-7714.13343 PMC7113057

[42] Feldheiser A Aziz O Baldini G. Enhanced recovery after surgery (ERAS) for gastrointestinal surgery, part 2: consensus statement for anaesthesia practice. Acta Anaesthesiol Scand 2016;60:289–334. 26514824 10.1111/aas.12651 PMC5061107

[43] Ye Z Chen J Shen T. Enhanced recovery after surgery (ERAS) might be a standard care in radical prostatectomy: a systematic review and meta-analysis. Ann Palliat Med 2020;9:746–58. 32389010 10.21037/apm.2020.04.03

[44] Wu X Liu L Zhou F. Meta-analysis for the evaluation of perioperative enhanced recovery after gynaecological surgery. Ginekol Pol 2022;93:896–903. 36621969 10.5603/GP.a2022.0064

[45] Li YW Li W Wang ST. The autonomic nervous system: a potential link to the efficacy of acupuncture. Front Neurosci 2022;16:1038945. 36570846 10.3389/fnins.2022.1038945 PMC9772996

[46] Ma N Li X Li Q. Electroacupuncture relieves visceral hypersensitivity through modulation of the endogenous cannabinoid system. Acupunct Med 2023;41:224–34. 35957508 10.1177/09645284221107699

[47] Que B Tu Q Shi J. Effects of transcutaneous electrical acupoint stimulation on systemic inflammatory response syndrome of patients after percutaneous nephrolithotomy: a randomized controlled trial. Evid Based Complement Alternat Med 2021;2021:5909956. 34422076 10.1155/2021/5909956 PMC8376454

[48] Bercik P Denou E Collins J. The intestinal microbiota affect central levels of brain-derived neurotropic factor and behavior in mice. Gastroenterology 2011;141:599–609. 21683077 10.1053/j.gastro.2011.04.052

